# Efficacy and Safety of Pafuramidine versus Pentamidine Maleate for Treatment of First Stage Sleeping Sickness in a Randomized, Comparator-Controlled, International Phase 3 Clinical Trial

**DOI:** 10.1371/journal.pntd.0004363

**Published:** 2016-02-16

**Authors:** Gabriele Pohlig, Sonja C. Bernhard, Johannes Blum, Christian Burri, Alain Mpanya, Jean-Pierre Fina Lubaki, Alfred Mpoo Mpoto, Blaise Fungula Munungu, Patrick Mangoni N’tombe, Gratias Kambau Manesa Deo, Pierre Nsele Mutantu, Florent Mbo Kuikumbi, Alain Fukinsia Mintwo, Augustin Kayeye Munungi, Amadeu Dala, Stephen Macharia, Constantin Miaka Mia Bilenge, Victor Kande Betu Ku Mesu, Jose Ramon Franco, Ndinga Dieyi Dituvanga, Richard R. Tidwell, Carol A. Olson

**Affiliations:** 1 Swiss Tropical and Public Health Institute, Pharmaceutical Medicine Unit, Swiss Centre for International Health, Basel, Switzerland; 2 Swiss Tropical and Public Health Institute, Pharmaceutical Medicine Unit, Swiss Centre for International Health, Basel, Switzerland; 3 Pharmacy & Clinical Pharmacology at the Division of Clinical Pharmacology, University of Basel, Basel, Switzerland; 4 Swiss Tropical and Public Health Institute, Medical Services and Diagnostic, Basel, Switzerland; 5 Swiss Tropical and Public Health Institute, Department of Medicines Research, Basel, Switzerland; 6 Programme Nationale de Lutte contre la Trypanosomiase Humaine Africaine, Kinshasa, Democratic Republic of the Congo; 7 Hôpital Evangélique de Vanga, Vanga, Province of Bandundu, Democratic Republic of the Congo; 8 Mission Hospital of Vanga, Vanga, Democratic Republic of Congo; 9 Centre Hospitalier Lisungi BDOM, Kinshasa, Democratic Republic of the Congo; 10 Clinique Damas Aleka, Libreville, Gabon; 11 Institut National de Recherche Biomédicale, Kinshasa, Democratic Republic of the Congo; 12 Zone de Santé de Djuma, Djuma, Democratic Republic of the Congo; 13 Zone de Santé de Mpayi, Mpay, Democratic Republic of Congo; 14 Instituto de Combate e de Controlo das Tripanossomíases, Luanda, Angola; 15 Management Sciences for Health, Juba, South Sudan; 16 Ministry of Health, Kinshasa, Democratic Republic of the Congo; 17 Programme des Maladies Tropicales Négligées, Ministère de la Santé Publique Kinshasa, Democratic Republic of the Congo; 18 World Health Organisation Geneva, Department of Control of Neglected Diseases, Geneva, Switzerland; 19 World Health Organization, Luanda, Angola; 20 University of North Carolina, Department of Pathology and Lab Medicine, School of Medicine, Chapel Hill, North Carolina, United States of America; 21 Sapphire Oak Consultants, LLC, Lindenhurst, Illinois, United States of America; Hospital Infantil de Mexico Federico Gomez, UNITED STATES

## Abstract

**Background:**

Sleeping sickness (human African trypanosomiasis [HAT]) is a neglected tropical disease with limited treatment options that currently require parenteral administration. In previous studies, orally administered pafuramidine was well tolerated in healthy patients (for up to 21 days) and stage 1 HAT patients (for up to 10 days), and demonstrated efficacy comparable to pentamidine.

**Methods:**

This was a Phase 3, multi-center, randomized, open-label, parallel-group, active control study where 273 male and female patients with first stage *Trypanosoma brucei gambiense* HAT were treated at six sites: one trypanosomiasis reference center in Angola, one hospital in South Sudan, and four hospitals in the Democratic Republic of the Congo between August 2005 and September 2009 to support the registration of pafuramidine for treatment of first stage HAT in collaboration with the United States Food and Drug Administration. Patients were treated with either 100 mg of pafuramidine orally twice a day for 10 days or 4 mg/kg pentamidine intramuscularly once daily for 7 days to assess the efficacy and safety of pafuramidine versus pentamidine. Pregnant and lactating women as well as adolescents were included.

The primary efficacy endpoint was the combined rate of clinical and parasitological cure at 12 months. The primary safety outcome was the frequency and severity of adverse events. The study was registered on the International Clinical Trials Registry Platform at www.clinicaltrials.gov with the number ISRCTN85534673.

**Findings/Conclusions:**

The overall cure rate at 12 months was 89% in the pafuramidine group and 95% in the pentamidine group; pafuramidine was non-inferior to pentamidine as the upper bound of the 95% confidence interval did not exceed 15%. The safety profile of pafuramidine was superior to pentamidine; however, 3 patients in the pafuramidine group had glomerulonephritis or nephropathy approximately 8 weeks post-treatment. Two of these events were judged as possibly related to pafuramidine. Despite good tolerability observed in preceding studies, the development program for pafuramidine was discontinued due to delayed post-treatment toxicity.

## Author Summary

Sleeping sickness, or human African trypanosomiasis (HAT), is a neglected tropical disease. Because only 2 treatment options are available to treat persons with stage 1 disease, and both require parenteral administration, oral drugs would be of great benefit to the affected population. In this Phase 3, multi-center, randomized, open-label, parallel-group study, we compared oral pafuramidine with intramuscular pentamidine in persons in sub-Sahara Africa with first stage HAT. At 12 months, the overall cure rates (combined clinical and parasitological cure) were similar: 89% in the pafuramidine group and 95% in the pentamidine group. At 24 months, the cure rates continued to be high: 84% and 89%, respectively. Pafuramidine’s safety profile was superior to the comparator drug, and it was consistent with the overall safety profile seen in previous Phase 2 studies. Upon further analysis, however, a renal safety issue was identified as being possibly related to pafuramidine and further clinical development was halted. Nevertheless, the clinical studies conducted in the pafuramidine development program provide a model for future studies in rural Africa.

## Introduction

Sleeping sickness (human African trypanosomiasis [HAT]) is a neglected tropical disease with limited treatment options that currently require parenteral administration. *Trypanosoma brucei (T*.*b*.*) gambiense* is found in 24 countries in west and central Africa and currently accounts for over 98% of reported cases [1]. Despite the long history of the disease (first cases reported in 1373/1374), the drugs available to treat it are toxic, difficult to administer, and stage-specific [2].

First stage symptoms entail bouts of fever, headaches, joint pains, and itching, and a person can be infected for months or even years without major signs or symptoms of the disease. When more evident symptoms emerge, the patient is often already in an advanced disease stage where the central nervous system is affected (second stage). The majority of current HAT research is focused on stage 2 of the disease, which requires drugs that can cross the blood-brain barrier. Drugs for stage 2 HAT are either too toxic (melarsoprol) or have too complex a regimen (nifurtimox-eflornithine combination treatment) for use against the first stage of the disease.

Only two drugs are approved for treatment of stage 1 HAT: pentamidine (only for *T*.*b*. *gambiense*) and suramin (only for *T*.*b*. *rhodesiense*), which are both administered parenterally. Suramin, synthesized as a dye in 1916 [3], has been used for the treatment of sleeping sickness since 1922 [4] [5], but it can cause undesirable effects in the urinary tract and allergic reactions [1]. Pentamidine, introduced in 1937, was developed as an analog of synthalin, a hypoglycemic agent with anti-trypanosomal activity [6] [7]. Pentamidine is administered by the intramuscular route and has a reported treatment failure rate after a course of five injections of approximately 7% [8] [9] [10]. Though this efficacy profile is encouraging, treatment with pentamidine has limitations. It requires injection, which hampers its use in rural treatment facilities, and though adverse reactions are usually reversible and its most serious long-term consequence, diabetes, is rare, the treatment is accompanied by a high frequency of adverse events, including hypotension, nephrotoxic effects, leukopenia, and hypo- and hyperglycemia [11] [12].

There is no vaccine for *T*.*b*. *gambiense* HAT and there is a great need for new safe and efficacious drugs that would be easy to use in rural health centers and affordable. In 2000, the promising orally administered pro-drug pafuramidine was chosen for clinical development by the Consortium for Parasitic Drug Development, which was founded in 1999 to foster the development of compounds with antiprotozoal activity [13]. Pafuramidine is the dimethoxime prodrug of furamidine (which has demonstrated excellent efficacy *in vitro* against *T*.*b*. *rhodesiense*) [14]. Pafuramidine was shown to be effective *in vivo* in the acute model (first stage disease) in mice [15] [16] and in monkeys (green vervet monkey [17] and rhesus monkey [18]). Preclinical evaluation *in vitro* as well as animal testing indicated no major safety concerns.

In 2000, pafuramidine was further evaluated in Phase 1 studies in healthy patients after single and multiple dosing (up to 21 days) and was well tolerated [19]. The subsequent Phase 2 studies (conducted from 2001 to 2007) in patients with stage 1 sleeping sickness supported this finding [20]. Efficacy after 5 days of treatment was limited; therefore, to attain efficacy comparable to that of pentamidine, the treatment duration was prolonged to 10 days. The pharmacokinetic properties of pafuramidine (in particular, the lack of proportional conversion of DB289 to DB75 at therapeutic doses) precluded using a higher dose to improve efficacy [19] [20] [21] [22].

This single, confirmatory, pivotal Phase 3 study was developed to support the registration of pafuramidine for treatment of stage 1 HAT under a Special Protocol Assessment in collaboration with the United States (US) Food and Drug Administration (FDA). The primary objective of the study was to demonstrate the non-inferiority of oral pafuramidine versus intramuscular pentamidine for treatment of first stage HAT caused by *T*.*b*. *gambiense*. Since safety and efficacy of a new drug should, if at all possible, be established in a study population representative of the target population, the secondary objective was to include pregnant and lactating women as well as adolescents in the study. Reproductive studies of pafuramidine in animals have not indicated any embryo or fetal toxicity or other effects on reproductive function of adult male and female rats or rabbits. Therefore, it was considered appropriate and was approved by the US FDA to proceed with studies including pregnant and lactating women.

## Methods

### Design

This was a multi-center, multi-country, open-label (sponsor-blinded), parallel-group, comparator-controlled, randomized Phase 3 study to compare the efficacy, safety, and tolerability of pafuramidine and pentamidine in 273 patients with first stage HAT caused by *T*.*b*. *gambiense*. The study was conducted at six African sites where *T*.*b*. *gambiense* sleeping sickness is endemic: two trypanosomiasis reference centers (Angola and the Democratic Republic of the Congo [DRC]) and four hospitals (1 in South Sudan and three in the DRC) from August 2005 (first patient enrolled) to September 2009 (last patient follow-up completed).

The study was registered on the International Clinical Trials Registry Platform at www.clinicaltrials.gov with the number ISRCTN85534673. International Protocol #289-C-006.

#### Ethics statement

This will certify that the Institutional Review Boards (IRBs) at the University of North Carolina at Chapel Hill, administered by the Office of Human Research Ethics, are organized and operate according to applicable laws and regulations governing research involving human subjects. These include, when applicable, statutes of the State of North Carolina and regulations of the Food and Drug Administration (21 CFR 50 and 56) and the Department of Health and Human Services [45 CFR 46 (the "Common Rule") and 45 CFR 164 (the Health Insurance Portability and Accountability Act, HIPPAA]. In addition, the IRBs conform, when applicable, to Good Clinical Practice (GCP) guidelines of the International Conference of Harmonization (ICH), to the extent these do not contradict DHHS and FDA regulations. The University of North Carolina at Chapel Hill holds a Federalwide Assurance, FWA 4801, approved by the federal Office for Human Research Protections (OHRP).

This study was approved by the following independent review boards and independent ethics committees: Ethikkommission beider Basel, Switzerland; the institutional review board of the University of North Carolina, School of Medicine, US., IRB# 05–2062 (Formerly 05-PATH/LAB-573; the Ethics Committee for Trypanosomiasis Research of the Ministry of Health of Angola; the Ethics Committee of the Ministry of Health of the DRC; and the Ethics Committee of the Ministry of Health of the Government of Southern Sudan.

Patients were randomized (1:1) to either 100 mg of pafuramidine orally twice a day (BID) for 10 days (n = 136) or 4 mg/kg pentamidine intramuscular injection once daily for 7 days (n = 137).

### Changes to Trial Design

There was one protocol amendment that provided detailed microscopy instructions for examining blood and CSF for the presence of trypanosomes and determining WBC count in CSF. The amendment also detailed randomization of pregnant and lactating women in a separate strata, and provided additional clarifications and administrative changes.

### Study Patients

Male and female patients were eligible to participate if they were ≥12 years of age, weighed ≥30 kg, had first stage *T*.*b*. *gambiense* infection documented by the presence of trypanosomes in the blood and/or lymph, and had no evidence of second stage disease (no trypanosomes detected in the cerebrospinal fluid [CSF] and ≤5 white blood cells [WBCs]/mm^3^ in CSF). Patients were also excluded if tested positive for malaria or helminth infections. Patients were not tested for HIV prior to treatment. Patients were treated at two trypanosomiasis reference centers (Angola and the DRC) and four hospitals (1 in South Sudan and three in the DRC). Written informed consent was obtained from each patient. If the patient was a minor or mentally impaired, a legal guardian also signed the consent form and if a patient was illiterate, an impartial witness assisted in the consent process.

Pregnant and lactating female patients as well as adolescents 12 to 15 years could be enrolled. Adolescents underwent additional safety laboratory testing at the 3-month post-treatment visit. Eligible pregnant and lactating female patients could participate with the understanding that additional safety measurements regarding course and outcome of the pregnancy and/or the health of their infant would be performed.

Patients were excluded if they had a possible or confirmed second stage *T*.*b*. *gambiense* infection (ie, presence of parasite in the CSF upon microscopic examination or a WBC count in the CSF of >5 mm^3^); any active, clinically relevant medical conditions that in the investigator’s opinion might jeopardize patient safety or interfere with study participation; presented with a score of less than 9 on the Glasgow Coma Scale; were previously treated for HAT; or displayed other conditions that would compromise participation.

### Interventions

Screening occurred within 7 weeks prior to dosing with pafuramidine or pentamidine (within 6 weeks prior to the baseline evaluation) using the card agglutination test for trypanosomes [11] [12] and microscopic examination (thin and/or thick smear) of blood and lymph node aspirate for trypanosomes either at the trypanosomiasis treatment centers or by mobile diagnostic units. All diagnostic tests performed by mobile teams were repeated in the treatment centers. Lumbar puncture was performed at the treatment centers in all trypanosome-positive cases detected by any method and the disease stage was determined by microscopic examination of CSF for trypanosomes and by WBC counts. If the result was negative, a blood sample was examined (including hematocrit centrifugation [23] and miniature anion exchange centrifugation technique [m-AECT] [24]). All patients were tested for malaria, and filaria using thick and thin blood smears and for diarrhea. If necessary, malaria treatment was given before enrolment; filariasis therapy was administered after study treatment when necessary. Patients were admitted as in-patients to the clinical site for the full duration of the treatment/observation period (11 days for pafuramidine or 7 days for pentamidine). Other baseline documentation included demographics, medical history, signs and symptoms of HAT, and concomitant disease(s) and medication(s).

Clinical supplies of pafuramidine were provided to the sites in bottles (50 tablets of 100 mg) labeled to indicate study drug, strength, expiration date, protocol number, and other information according to local regulations. Pentamidine was provided locally by the agency (generally the national HAT control programs) responsible for each center in the form of pentamidine isethionate for injection in single-dose vials at 200 mg/vial.

For efficacy assessments, patients underwent microscopic examination of blood (thin and/or thick smear), hematocrit centrifugation of blood [25], microscopic examination of lymph node aspirate, and microscopic examination of blood after m-AECT concentration at the end of treatment and at 3, 6, 12, and 18 months post-treatment [26]. Lumbar puncture was performed for microscopic examination of CSF fluid for WBCs and trypanosomes at baseline and 6, 12, and 18 months post-treatment, and at any other evaluation where relapse was suspected or trypanosomes were demonstrated in blood or lymph nodes. Additional assessments of clinical efficacy were performed at 24 months post-treatment.

During the treatment and post-treatment period, safety evaluations included vital signs, physical examination, adverse event monitoring, laboratory tests, electrocardiogram (ECG) monitoring to the extent possible at each site, and documentation of concomitant medications. Signs or symptoms of HAT were queried and graded. Laboratory tests assessed clinical chemistry (aspartate aminotransferase, alanine aminotransferase, total bilirubin, glucose, and creatinine) and hemoglobin. Electrocardiograms were performed at baseline, 1 hour prior to dosing, and 1 hour after dosing for all patients. An additional ECG was obtained on Day 7 post-treatment for pafuramidine-treated patients.

### Outcomes

Clinical response definitions are listed in Table 1. The primary efficacy endpoint was the combined rate of cure and probable cure at the 12-month follow-up in the per-protocol data set. The overall cure rate was defined as the proportion of treated patients with no clinical signs and symptoms of HAT, no evidence of trypanosomes in any body fluid examined, and no treatment with other trypanocidal agents for any reason; in addition, ≤5 WBCs/mm^3^ in CSF obtained from a lumbar puncture was required.

**Table 1:** Clinical response definitions.

Category	WHO Term	Characteristics
**Parasitological cure**	Cure	Lumbar puncture performed: no evidence for parasitological relapse and ≤5 WBCs/mm^3^ in CSF
**Clinical cure**	Probable cure	No evidence for parasitological relapse in absence of lumbar puncture (no clinical signs; symptoms/signs attributable to other disease; investigator decides no retreatment necessary)
		or No parasitological evidence of relapse with 6–0 WBCs/mm^3^ in CSF
		Action: No retreatment
**Probable relapse**	Probable relapse	No evidence of parasitological relapse and >20 WBCs/mm^3^ in CSF
		or No evidence of parasitological relapse in a patient who refuses lumbar puncture and who presents with clinical signs of HAT and/or marked deterioration of clinical condition relative to previous evaluations that is unlikely due to another disease. In addition, in the investigator’s opinion, all other reasons for the patient’s clinical status have been excluded and rescue treatment is required.
		Action: Retreatment
**Relapse**	Relapse	Trypanosomes have been detected in any body fluid
		Action: Retreatment
**Death**	Death	Death of patient during treatment or follow-up; death will be categorized based on likely or definite cause of death as HAT; adverse event related to treatment of HAT; causes unrelated to HAT and treatment; unknown causes

CSF = cerebrospinal fluid, HAT = human African trypanosomiasis, WBCs = white blood cells, WHO = World Health Organization

Secondary efficacy endpoints were cure, clinical cure, probable relapse, relapse, and death rates at the end of treatment and all follow-up visits. Parasitological cure, probable relapse, relapse, and death rates were also assessed at the 12-month test of cure evaluation and at the 24-month evaluation; the clinical cure was considered equivalent to the parasitological cure at the 24-month evaluation.

Study efficacy parameters and timing of post-treatment evaluations were based on WHO recommendations for clinical product development for HAT [27]. Although 18 months post-treatment is recommended to assess clinical cure in HAT control programs due to anticipated increased drop-out rates from follow-up after 6 to 12 months, the 12-month evaluation was chosen as the primary endpoint in this study in order to maintain a robust data set for the primary analysis.

Safety was assessed through the end of treatment evaluation and included adverse events, laboratory results, vital sign measurements, physical examinations, and use of any concomitant medications. The term “adverse event” included any of the following events that developed or increased in severity during the study: 1) any signs or symptoms whether thought to be related or unrelated to HAT; 2) any clinically significant laboratory abnormality; or 3) any abnormality detected during physical examination. Adverse events were graded by the investigator (1 = mild, 2 = moderate, 3 = severe, 4 = intolerable). Adverse events were assessed at every study visit and were classified according to the terms found in the Medical Dictionary for Regulatory Activities (MedDRA).

A serious adverse event was defined as any event that suggested a significant hazard, contraindication, side effect, or precaution, it included any event that: 1) is fatal; 2) is life threatening; 3) is a persistent or significant disability/incapacity; 4) requires or prolongs in-patient hospitalization; 5) is a congenital anomaly/birth defect; or 6) is an important medical event, based upon appropriate medical judgment, that may jeopardize the patient or may require medical or surgical intervention to prevent one of the other outcomes defining serious.

### Changes to Outcomes

There were no changes to any of the outcomes.

### Sample Size

A total of 250 patients, 125 patients per treatment group, were originally expected to be treated in order to include 100 patients per treatment group in the per-protocol. This sample size provided more than 90% power to demonstrate non-inferiority of pafuramidine to pentamidine for the primary endpoint, when the study drugs have equivalent probable cure rates of 95% in the per-protocol analysis. Non-inferiority comparison was conducted with an alpha equal to 0.048 and non-inferiority margin (ie, delta) of 0.15.

### Stopping Guidelines and Interim Analysis

The sponsor may have terminated this study prematurely, either in its entirety or at a particular site, for reasonable cause or safety concerns. The sponsor remained blinded and the data were provided to the data safety monitoring board (DSMB) for evaluation. Based on these data, the DSMB made recommendations to the sponsor regarding continuation of the study. The study could have been stopped if: 1) any new untoward safety issues were identified in the pafuramidine treatment group such that pafuramidine was significantly less safe than pentamidine; 2) the re-estimated sample size exceeded 500 patients to achieve 90% power for the primary efficacy endpoint; or 3) efficacy analysis indicated that pafuramidine was significantly more effective than pentamidine (p<0.002).

An interim analysis was to be conducted when half of the enrolled patients reached the 12-month post-treatment endpoint, however, this was not done because the pafuramidine development program was discontinued due to delayed post-treatment toxicity (details are provided in the Harms section).

### Randomization

Patients were randomly assigned by the local investigators to receive either pafuramidine or pentamidine in the order in which they were enrolled. Randomization was carried out in blocks of variable size following a randomization schedule prepared by the sponsor; randomization of pregnant and lactating women was stratified. Each study site was provided with series of individual envelopes each containing a card with the treatment assignment for 1 patient and a control number. After a patient signed the informed consent and inclusion/exclusion criteria were confirmed, the investigator opened the next envelope in the randomization list to obtain treatment assignment for that patient and then transferred the control number to the patient’s case report form.

### Blinding

The study was open-label, since pafuramidine is administered orally and pentamidine is administered intramuscularly. However, the sponsor was blinded to treatment assignments.

### Statistical Methods

The primary efficacy analysis, demonstrating the non-inferiority of pafuramidine to pentamidine for the combined rate of cure and probable cure, was conducted with an alpha equal to 0.048 and non-inferiority margin (ie, delta) of 0.15. The comparison was made with a 1-sided 97.6% confidence interval (CI) for the treatment difference in parasitological cure rate. The normal approximation to the binomial distribution with continuity correction was used to construct the CI. The primary data set for efficacy analysis was the per-protocol data set.

The efficacy analysis was carried out for the per-protocol data set (primary analysis), the intention-to-treat (ITT) data set, and the modified ITT (mITT) data set (supportive analyses). The per-protocol data set was defined as patients who received a minimum of 7 days of pafuramidine or five injections of pentamidine and who attended the test of cure assessment at Month 12 or reached an efficacy endpoint (death, non-response, or relapse) at an earlier time. Patients without lumbar puncture at Month 12 were included and their outcome was assessed based on clinical signs and symptoms and parasitological findings from any body fluid examined.

The mITT data set consisted of all patients who received the minimum amount of randomized study drug (7 or 5 days) and for whom an end-of-treatment assessment and at least one follow-up efficacy assessment were available. Patients who had received at least one dose of study drug were included in the ITT data set and patients who were lost to follow-up or discontinued from the study for any reason were considered treatment failures in the ITT analysis. The last observation carried backward was used to account for missing data at an earlier evaluation (in case of cure at a later evaluation). For the mITT analysis, missing data were addressed according to the last observation carried forward principle.

The secondary efficacy variables were summarized at all time points with point estimates and 1-sided 97.5% CIs for the difference between treatments.

The safety data set consisted of all patients who received at least one dose of study drug and had at least one safety evaluation after dosing. Treatment group differences in the proportion of patients who reported treatment-emergent adverse events for Day 1 through Day 11 were assessed with Fisher’s exact test.

The number and percentage of patients who reported treatment-emergent adverse events were summarized for each treatment group at the system organ class, high-level group terms, and preferred term level. Treatment group differences in the proportion of patients who reported each high-level group term were assessed with Fisher’s exact test.

Any clinically significant physical examination changes from baseline were captured as an adverse event.

## Results

### Participant Flow

**Fig 1:**
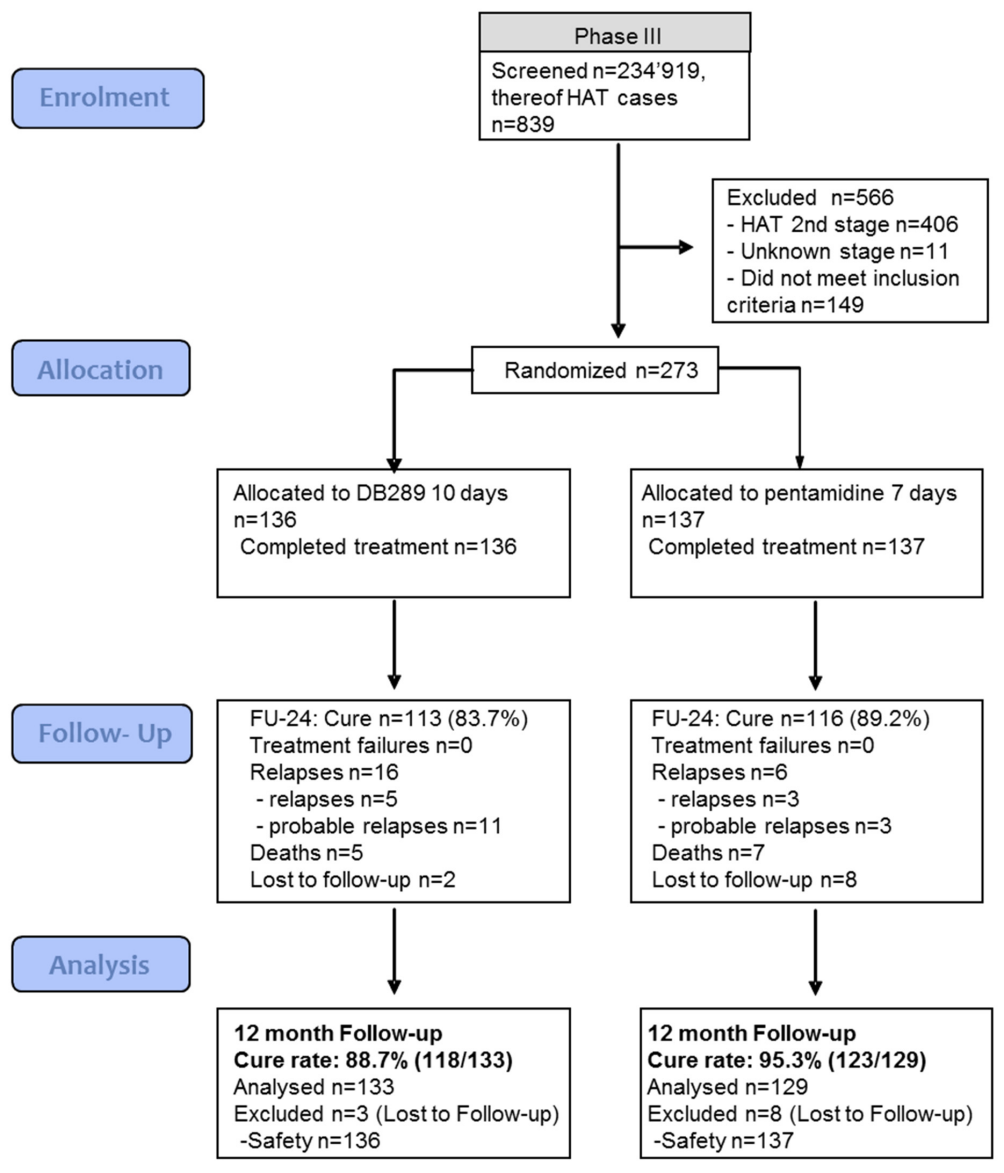
Pafuramidine (DB289) Phase 3 study CONSORT flowchart.

First stage HAT patients rarely present at a hospital or a treatment center. Therefore, intense screening activities were necessary. Between July 2005 and March 2007, a total of 234,919 patients were screened to find 839 individuals affected with HAT (Fig 1). The exclusion rate was high (566 of 839 patients, 67.5%); primary reasons were that patients had stage 2 HAT and did not meet inclusion criteria. A total of 273 patients were randomized: 136 patients received pafuramidine and 137 received pentamidine; all 273 completed the study. Most of the patients (91%) were enrolled in the DRC (248 of 273 patients); 5.5% were enrolled in Angola (15 of 273 patients), and 3.7% (10 of 273 patients) were enrolled in South Sudan.

As shown in Fig 1, follow-up attendance at Month 24 was good; only 2 patients in the pafuramidine group and 8 patients in the pentamidine group were lost to follow-up.

### Baseline Data

As seen in Table 2, baseline demographic characteristics between the two treatment groups were similar. The median age of patients in both treatment groups was approximately 30 years, and the majority of patients in both groups were female (70% and 66%, respectively).

**Table 2:** Baseline demographics.

	Pafuramidine 100 mg BID (N = 136) n (%)	Pentamidine 4 mg/kg QD (N = 137) n (%)
**Age (years)**		
Mean (SD)	33.4 (13.66)	33.7 (14.10)
Median	30	31
Min, max	12, 64	13, 75
n (%) >64 years	0	5 (3.6)
**Sex**		
n (%) male	41 (30.1)	47 (34.3)
n (%) female	95 (69.9)	90 (65.7)
**Status**		
Adolescents	8 (5.9)	8 (5.8)
Pregnant	4 (2.9)	8 (5.8)
Lactating	28 (20.6)	26 (19.0)
Pregnant & lactating	1 (0.7)	1 (0.7)
**Weight**		
Mean (SD)	44.7 (7.90)	45.7 (7.82)
Median	44	46
Min, max	30, 64	30, 69
**Height (cm)**	N = 135	N = 137
Mean (SD)	160.4 (9.41)	160.6 (9.38)
Median	160	160
Min, max	139, 187	138, 185

BID = twice a day, QD = once a day, SD = standard deviation

### Numbers Analyzed and Excluded

As shown in Fig 1, 133 of 136 patients (97.8%) in the pafuramidine group and 129 of 137 patients (94.2%) in the pentamidine group were included in the efficacy analysis. Three patients in the pafuramidine group and 8 patients in the pentamidine group were excluded because they were lost to follow-up.

### Outcomes and Estimations

As shown in Table 3 at the test of cure evaluation (12 months post-treatment), the combined rate of cure and probable cure was 89% (118 of 133 patients) in the pafuramidine group and 95% (123 of 129 patients) in the pentamidine group in the per-protocol population.

**Table 3:** Combined rate of cure and probable cure at 12 months.

Patient Group	Pafuramidine	Pentamidine	95% CI^a^
	100 mg BID	4 mg/kg QD	
	n/N (%)	n/N (%)	
**Per-protocol population**	118/133 (88.7)	123/129 (95.3)	-0.1, 13.1
**ITT population**	118/136 (86.8)	123/137 (89.8)	-4.6, 10.6
**mITT population**	121/136 (89.0)	131/137 (95.6)	0.4, 12.9

BID = twice a day, CI = confidence interval, ITT = intent-to-treat population, mITT = modified intent-to-treat population, QD = once a day

^a^ Confidence intervals are based on normal approximation to the binomial for pentamidine–pafuramidine.

Pafuramidine was non-inferior to pentamidine as the upper bound of the 95% CI did not exceed 15%. This finding was supported by the 24-month follow-up data, where cure rates of 84% for the pafuramidine group and 89% for the pentamidine group were maintained (Table 4). Supportive analysis in the ITT and mITT populations generated similar results.

**Table 4:** Combined rate of cure and probable cure at 24 months.

	Pafuramidine	Pentamidine	95% CI^a^
	100 mg BID	4 mg/kg QD	
	n/N (%)	n/N (%)	
**Per-protocol population**	113/135 (83.7)	116/130 (89.2)	-2.7, 13.7
**ITT population**	113/136 (83.1)	116/137 (84.7)	-7.1, 10.3
**mITT population**	114/136 (83.8)	123/137 (89.8)	-2.0, 14.0

BID = twice a day, CI = confidence interval, ITT = intent-to-treat population, mITT = modified intent-to-treat population, QD = once a day

^a^ Confidence intervals based on normal approximation to the binomial for pentamidine–pafuramidine

Table 5 summarizes the secondary efficacy variables for each follow-up visit, including the cumulative number of cures, probable cures, probable relapses, relapses, and deaths for each treatment group. There were no deaths in either treatment group during the active study period, and all patients responded to treatment. Relapses in the pafuramidine treatment group appeared to be evenly distributed over the whole follow-up period, whereas a trend for late relapses was observed in the pentamidine treatment group.

**Table 5:** Secondary efficacy variables at months 3, 6, 12, 18, and 24 (per-protocol dataset).

	Pafuramidine	Pentamidine	
WHO Efficacy Criteria	100 mg BID	4 mg/kg QD	
Time Point	n/N (%)	n/N (%)	95% CI^a^
**Cure**			
Month 3	2/135 (1.5)	1/137 (0.7)	-3.2, 1.7
Month 6	124/135 (91.9)	131/137 (95.6)	-2.0, 9.5
Month 12	117/133 (88.0)	122/129 (94.6)	-0.2, 13.4
Month 18	99/131 (75.6)	109/129 (84.5)	-0.7, 18.6
Month 24	86/135 (63.7)	83/130 (63.8)	-11.4, 11.7
**Probable cure**			
Month 3^b^	131/135 (97.0)	135/137 (98.5)	-2.0, 5.0
Month 6^b^	5/135 (3.7)	3/137 (2.2)	-5.5, 2.5
Month 12^b^	4/133 (3.0)	4/129 (3.1)	-4.1, 4.3
Month 18^b^	16/131 (12.2)	13/129 (10.1)	-9.8, 5.5
Month 24^c^	28/135 (20.7)	34/130 (26.2)	-4.8, 15.6
**Probable relapse**			
Month 3	0	0	--
Month 6	2/135 (1.5)	1/137 (0.7)	-3.2, 1.7
Month 12	5/133 (3.8)	1/129 (0.8)	-6.6, 0.6
Month 18	7/131 (5.3)	1/129 (0.8)	-8.7, -0.4
Month 24	11/135 (8.1)	3/130 (2.3)	-11.1, -0.6
**Relapse**^**d**^			
Month 3	2/135 (1.5)	1/137 (0.7)	-3.2, 1.7
Month 6	2/135 (1.5)	1/137 (0.7)	-3.2, 1.7
Month 12	3/133 (2.3)	1/129 (0.8)	-4.4, 1.5
Month 18	4/131 (3.1)	3/129 (2.3)	-4.7, 3.2
Month 24	5/135 (3.7)	3/130 (2.3)	-5.5, 2.7
**Death**			
Month 3	0	0	--
Month 6	2/135 (1.5)	1/137 (0.7)	-3.2, 1.7
Month 12	4/133 (3.0)	1/129 (0.8)	-5.5, 1.0
Month 18	5/131 (3.8)	3/129 (2.3)	-5.7, 2.7
Month 24^e^	5/135 (3.7)	7/130 (5.4)	-3.3, 6.7

BID = twice a day, CI = confidence interval, QD = once a day, WHO = World Health Organization

^a^ Confidence intervals based on normal approximation to the binomial.

^b^ Includes cases of “uncertain evolution” [27]

^c^ Includes cases classified as probable cure based on oral information

^d^ One patient in the pentamidine group was classified as a treatment failure at end of treatment and is included in Relapse at Month 3.

^e^ One death was reported in the pafuramidine group subsequent to the last patient visit and is not included in the efficacy endpoints.

The numbers of adolescents and pregnant women (8 and 10, respectively) were too small to make any definitive conclusions about efficacy in these patients. For lactating women, the overall cure rates of pafuramidine and pentamidine at the test of cure evaluation were the same (23 of 26 patients [88.4%] in each group). However, the low number of lactating women also did not allow for definitive conclusions.

### Harms

No patients prematurely discontinued due to an adverse event during the study.

As shown in Table 6, the most commonly reported adverse events were injection site pain, pyrexia, hypoglycemia, and hypotension. These events occurred more frequently in the pentamidine group than the pafuramidine group, with the exception of pyrexia, which occurred more frequently in the pafuramidine group. The incidence of patients with at least one adverse event overall for Day 1 through Day 11 was statistically significantly less in the pafuramidine treatment group (82%, 111 of 136) than in the pentamidine group (99%, 135 of 137) (p<0.05). Among high-level group terms, there were statistically significant differences in favor of the pafuramidine treatment group versus the pentamidine group for hepatobiliary investigations (7% vs. 77%, respectively); renal and urinary tract investigations and urinalyses (2% vs. 9%, respectively); glucose metabolism disorders (including diabetes mellitus) (6% vs. 18%, respectively); and decreased nonspecific blood pressure disorders and shock (44% vs. 62%, respectively) (p<0.05 for all).

**Table 6:** Treatment-emergent adverse events experienced by ≥5% of patients in either treatment group (treatment period).

	Pafuramidine 100 mg BID		Pentamidine 4 mg/kg QD	
SOC	(N = 136)		(N = 137)	
HLGT	n (%)		n (%)	
Preferred Term	All Adverse Events	Treatment-Related Adverse Events^a^	All Adverse Events	Treatment-Related Adverse Events^a^
**Total patients with at least 1 adverse event^b^**	111 (81.6)	54 (39.7)	135 (98.5)	127 (92.7)
**Gastrointestinal disorders**	21 (15.4)	10 (7.3)	23 (16.8)	12 (8.8)
Gastrointestinal signs and symptoms	18 (13.2)	9 (6.6)	19 (13.9)	12 (8.8)
Abdominal pain	7 (5.1)	1 (0.7)	4 (2.9)	2 (1.5)
Nausea	4 (2.9)	2 (1.5)	11 (8.0)	9 (6.6)
**General disorders and administration site conditions**	46 (33.8)	10 (7.4)	61 (44.5)	41 (29.9)
Administration site reactions	0	0	35 (25.5)	35 (25.5)
Injection site pain	0	0	33 (24.1)	33 (24.1)
Body temperature conditions	43 (31.6)	8 (5.9)	33 (24.1)	10 (7.3)
Pyrexia	42 (30.9)	8 (5.9)	31 (22.6)	10 (7.3)
**Investigations**	33 (24.3)	10 (7.4)	111 (81.0)	108 (78.8)
Haematology investigations (incl blood groups)	17 (12.5)	5 (3.7)	11 (8.0)	6 (4.4)
Haemoglobin decreased	7 (5.1)	2 (1.5)	6 (4.4)	3 (2.2)
Haemoglobin increased	10 (7.4)	3 (2.2)	5 (3.6)	3 (2.2)
Hepatobiliary investigations^b^	10 (7.4)	4 (2.9)	106 (77.4)	105 (76.6)
Alanine aminotransferase increased	2 (1.5)	2 (1.5)	72 (52.6)	71 (51.8)
Aspartate aminotransferase increased	7 (5.1)	4 (2.9)	104 (75.9)	103 (75.2)
Metabolic, nutritional and blood gas investigations	9 (6.6)	3 (2.2)	13 (9.5)	10 (7.3)
Blood glucose decreased	5 (3.7)	2 (1.5)	12 (8.8)	10 (7.3)
Renal and urinary tract investigations and urinalyses^b^	3 (2.2)	0	12 (8.8)	6 (4.4)
Blood creatinine increased	3 (2.2)	0	11 (8.0)	5 (3.6)
**Metabolism and nutrition disorders**	11 (8.1)	1 (0.7)	30 (21.9)	24 (17.5)
Glucose metabolism disorders (including diabetes mellitus)^b^	8 (5.9)	0	25 (18.2)	21 (15.3)
Hypoglycaemia	8 (5.9)	0	25 (18.2)	21 (15.3)
**Nervous system disorders**	24 (17.6)	8 (5.9)	26 (19.0)	19 (13.9)
Headaches	19 (14.0)	5 (3.7)	13 (9.5)	4 (2.9)
Headache	19 (14.0)	5 (3.7)	13 (9.5)	4 (2.9)
Neurological disorders NEC	9 (6.6)	4 (2.9)	16 (11.7)	15 (10.9)
Dizziness	9 (6.6)	4 (2.9)	6 (4.4)	5 (3.6)
Dysgeusia	0	0	11 (8.0)	11 (8.0)
**Skin and subcutaneous tissue disorders**	7 (5.1)	2 (1.5)	1 (0.7)	0
Epidermal and dermal conditions^2^	7 (5.1)	2 (1.5)	1 (0.7)	0
**Vascular disorders**	64 (47.1)	29 (21.3)	86 (62.8)	79 (57.7)
Decreased and nonspecific blood pressure disorders and shock^2^	60 (44.1)	28 (20.6)	85 (62.0)	78 (56.9)
Hypotension	60 (44.1)	28 (20.6)	85 (62.0)	78 (56.9)

BID = twice a day, HLGT = high-level group terms, NEC = not elsewhere classified, QD = once a day, SOC = system organ class

^a^ Considered at least possibly related to study drug by the Investigator.

^b^ Statistically significant (p<0.05) difference between treatment groups for overall incidence based on Fisher’s exact test.

A statistically significantly greater percentage of pafuramidine patients than pentamidine patients experienced epidermal and dermal conditions (5% vs. 1%, respectively) (p<0.05).

The majority of the adverse events were mild or moderate in severity and typical for patients recovering from first stage HAT.

The ECG results from this study were included in a separately published study on cardiac alterations in HAT [28]. In brief, the mean PQ and QTc intervals did not increase during treatment of first stage disease in either treatment group. The appearance and disappearance of repolarization changes at the end of treatment were comparable between groups.

A total of 43 patients experienced serious adverse events during the study (including the follow-up period): 19 of 136 patients (14.0%) in the pafuramidine group and 24 of 137 patients (17.5%) in the pentamidine group. Of these, only 3 patients had serious adverse events while on treatment: 1 in the pafuramidine group (cellulitis considered probably not related to study drug) and 2 in the pentamidine group (hypersensitivity considered not related to study drug and subcutaneous abscess considered probably related to the study drug). All other serious adverse events occurred during the follow-up period.

Of the 43 patients with serious adverse events, it was initially considered probable that only one was related to the study drug (subcutaneous abscess in the pentamidine group). Re-evaluation of the relatedness of these events to the study drug was subsequently performed when serious renal and hepatic post-treatment toxicity was observed in 3 patients in a supportive Phase 1 study of pafuramidine, which was conducted in South Africa (in December 2007), during the follow-up period of the current Phase 3 study. The Phase 1 study included 175 male and female volunteers taking oral pafuramidine 100 mg BID for 14 days [19]. Retrospectively, the glomerulonephritis reported for the 2 pafuramidine patients in the current Phase 3 study was considered to be possibly related to the study drug.

Thirteen patients (6 in the pafuramidine group and 7 in the pentamidine group) died during the follow-up period. All deaths were considered not related or probably not related to the study drug. Two deaths in the pafuramidine group were considered to be related to HAT; one death was considered to be related to relapse of HAT and another was considered to be associated with treatment of a HAT relapse with melarsoprol.

Safety data for adolescent as well as pregnant and lactating women were similar to the observations in the general population.

## Discussion

### Limitations

Although this study was conducted in rural conditions in Angola, South Sudan, and the DRC with local teams that had limited experience in clinical studies, this study was fully compliant with Good Clinical Practice and regulatory standards. In addition, this was the first Phase 3 study of a new drug intended for treatment of sleeping sickness conducted under a US FDA Investigational New Drug Application that followed contemporary International Conference on Harmonisation guidance.

### Interpretation

The results demonstrate the efficacy of pafuramidine in the treatment of first stage HAT, with an overall cure rate that was statistically non-inferior to that observed for pentamidine (89% vs. 95%, respectively) at 12 months post-treatment. The results obtained in the per-protocol set were confirmed in the ITT and mITT analysis populations, which included missing follow-up visits and patients lost to follow-up. The Month 24 results in all populations corroborate the 12-month results and demonstrate the robustness of the primary efficacy analysis.

Compared with patients who received pentamidine, pafuramidine-treated patients (total population including the subpopulations of adolescents and pregnant and lactating women) had lower rates of treatment-related adverse events (93% vs. 40%, respectively) and lower rates of adverse events related to hepatic, renal, and metabolic toxicity. An ECG analysis revealed no cardiotoxicity for either drug [28]. These data are consistent with the good tolerability observed for pafuramidine in the previous Phase 2 studies [20].

This study was also designed to evaluate efficacy and safety of pafuramidine and pentamidine in subpopulations that are particularly vulnerable to the long-term socioeconomic burdens associated with HAT, mainly pregnant and lactating women. However, the number of participating pregnant and lactating women was too low for a thorough separate analysis. The low number may be a result of social pressures and fear of treatment, which could be a detriment to seeking HAT treatment and going to a hospital. Low fertility and amenorrhea, which are often associated with HAT, may also have contributed [29]. From the limited number of relevant patients, there was no evidence for reduced efficacy or additional safety issues relating to pafuramidine compared with those observed in the overall study population. Numeric cure rates were similar and no specific safety issues were identified.

The initial safety profile observed for pafuramidine in this study was consistent with the results of preceding studies in the pafuramidine clinical development program [19] [20]. However, 3 patients in the pafuramidine group exhibited glomerulonephritis or nephropathy approximately 8 weeks post-treatment. On further examination, these events appeared to be similar to events that occurred in the previously mentioned supportive South African Phase 1 safety study. After re-evaluation, 2 of the 3 patients were considered to have events that were possibly related to pafuramidine by the principal investigator.

It should be noted that the analysis of safety data, particularly of the serious adverse events that occurred in the HAT study reported here, did not reveal any apparent negative long-term effects of pafuramidine. The patients who experienced renal toxicity recovered without sequelae, and the additional safety data obtained during the follow-up revealed no differences in abnormal biochemistry values between pafuramidine and pentamidine groups.

Eventually, clinical development of pafuramidine was discontinued in early 2008, since the renal toxicity observed in the additional Phase 1 study was considered to be an unacceptable risk. Preliminary evidence of the involvement of the kidney injury molecule (KIM-1) was only very recently provided through the use of a mouse diversity panel [30].

### Generalizability

From the perspective of study design, it is noteworthy that the 12-month endpoint for efficacy effectively predicted the clinical outcomes determined at the 24-month evaluation. Thus, 12 months is a meaningful endpoint for a sleeping sickness study with adequately performed follow-up. The infrastructure and technical expertise developed during the Phase 2 development program for pafuramidine were effectively leveraged to guide the screening, enrolment, and oversight of the larger study population included in this Phase 3 registration study, and eventually led to a unique and comprehensive data set. The successful conduct of the study was evidenced by the retention of 97% (265 of 273 patients) of the randomized patients at the 24-month (end-of-study) evaluation. Finally, the lessons learned from the Phase 2 development program were also helpful in ensuring that the Phase 3 study complied with Good Clinical Practice and regulatory standards required for a registration study [20].

The repeated success of clinical study conduct throughout the pafuramidine development program provides a model for future studies in rural Africa and will undoubtedly contribute to continued improvement of HAT control in sub-Saharan Africa.

## Supporting Information

S1 File CONSORT checklist. (DOC)
